# Tying eHealth Tools to Patient Needs: Exploring the Use of eHealth for Community-Dwelling Patients With Complex Chronic Disease and Disability

**DOI:** 10.2196/resprot.3500

**Published:** 2014-11-26

**Authors:** Carolyn Steele Gray, Daniel Miller, Kerry Kuluski, Cheryl Cott

**Affiliations:** ^1^Bridgepoint Collaboratory for Research and InnovationBridgepoint Active HealthcareToronto, ONCanada; ^2^Health System Performance Research NetworkInstitute of Health Policy, Management and EvaluationUniversity of TorontoToronto, ONCanada; ^3^Department of Physical TherapyUniversity of TorontoToronto, ONCanada

**Keywords:** eHealth, primary health care, patient-centered care, chronic disease, multimorbidity

## Abstract

**Background:**

Health policy makers have recently shifted attention towards examining high users of health care, in particular patients with complex chronic disease and disability (CCDD) characterized as having multimorbidities and care needs that require ongoing use of services. The adoption of eHealth technologies may be a key strategy in supporting and providing care for these patients; however, these technologies need to address the specific needs of patients with CCDD. This paper describes the first phase of a multiphased patient-centered research project aimed at developing eHealth technology for patients with CCDD.

**Objective:**

As part of the development of new eHealth technologies to support patients with CCDD in primary care settings, we sought to determine the perceived needs of these patients with respect to (1) the kinds of health and health service issues that are important to them, (2) the information that should be collected and how it could be collected in order to help meet their needs, and (3) their views on the challenges/barriers to using eHealth mobile apps to collect the information.

**Methods:**

Focus groups were conducted with community-dwelling patients with CCDD and caregivers. An interpretive description research design was used to identify the perceived needs of participants and the information sharing and eHealth technologies that could support those needs. Analysis was conducted concurrently with data collection. Coding of transcripts from four focus groups was conducted by 3 authors. QSR NVivo 10 software was used to manage coding.

**Results:**

There were 14 total participants in the focus groups. The average age of participants was 64.4 years; 9 participants were female, and 11 were born in Canada. Participants identified a need for open two-way communication and dialogue between themselves and their providers, and better information sharing between providers in order to support continuity and coordination of care. Access issues were mainly around wait times for appointments, challenges with transportation, and costs. A visual depiction of these perceived needs and their relation to each other is included as part of the discussion, which will be used to guide development of our eHealth technologies. Participants recognized the potential for eHealth technologies to support and improve their care but also expressed common concerns regarding their adoption. Specifically, they mentioned privacy and data security, accessibility, the loss of necessary visits, increased social isolation, provider burden, downloading responsibility onto patients for care management, entry errors, training requirements, and potentially confusing interfaces.

**Conclusions:**

From the perspective of our participants, there is a significant potential for eHealth tools to support patients with CCDD in community and primary care settings, but we need to be wary of the potential downfalls of adopting eHealth technologies and pay special attention to patient-identified needs and concerns. eHealth tools that support ongoing patient-provider interaction, patient self-management (such as telemonitoring), and provider-provider interactions (through electronic health record integration) could be of most benefit to patients similar to those in our study.

## Introduction

Health systems globally are shifting attention towards examining high users of the health system. In Ontario, Canada, only 1% of the province’s population accounts for 34% of costs, while 10% accounts for 79% of total system-wide costs [1]. Similar trends are also found in British Columbia, Canada [2], and in the United States [3]. The small group of high users includes a number of subpopulations; among them are patients with complex chronic disease and disability (CCDD). Patients with CCDD can be characterized as having multimorbidity (having two or more chronic illnesses) [4] and symptoms that have an impact on their daily living [5], which results in their using more care [1,6,7], experiencing poor care coordination [8], and having a higher risk of poor health outcomes than those with single illnesses only [7,9]. Biology and disease profile, however, capture only the chronic disease and disability aspect of CCDD. The complexity aspect requires attention to broader social, environmental, and contextual issues that have an impact on the health care needs of these patients, leading some to call for patient-centered approaches to care delivery [4].

Patient-centered care requires a “focus on the patient’s experience of illness and health care and on the systems that work to meet individual patients’ needs” (p. 48 [10]). A patient-centered approach to care requires focus at multiple levels. At the patient-provider level, patient-centered care involves communication, respect for patients, shared responsibility between patients and providers, access to information and education for patients and families, and support for the whole patient (ie, from a bio-psychosocial perspective). At the system level, patient-centered approaches require organizations and systems that place the patient at the center of care with particular attention to coordination, integration, and continuity of care [10-14]. eHealth technologies may be a key strategy to supporting patient-centered care through their ability to support improved access, continuity, communication, shared decision-making, and patient self-management [15-19].

While there have been many advances in adopting eHealth technologies to support chronic disease patients in hospital settings [20] and primary care settings [21], many of these tools are disease specific and may not be able to address the needs of patients with CCDD. We sought to address this gap by developing a suite of eHealth mobile apps and tools for use in team-based primary care settings to support patients with CCDD living in the community. In our broader project, we used a design evaluation approach that involves refining designs based on prior research and ongoing evaluation that involves end-users throughout the process [22]. In this paper, we report on the first stage in our development process in which we use an interpretive descriptive qualitative methodology to identify the perceived needs of community-dwelling patients with CCDD with respect to (1) the kinds of health and service issues that are important to them, (2) the information that should be collected and how it could be collected in order to help meet their needs, and (3) their views on the challenges/barriers to using eHealth mobile apps to collect the information.

## Methods

### Research Design

An interpretive description approach [23,24] was used to guide our study design and analysis method. Interpretive description, which comes from qualitative nursing research, aims to describe and interpret a “shared health or illness phenomenon from the perspective of those who live it” (p. 171 [23]). Given our intention to better understand the perceived needs of community-dwelling patients with CCDD, and the information sharing and eHealth technologies that could support those needs, an interpretive descriptive design was determined to be an appropriate approach.

### Context

Focus group participants were recruited from a Family Health Team (FHT)—an interprofessional primary care delivery model [25] in Ontario, Canada. The practice serves over 5000 people from the Riverdale community of Toronto as well as the Greater Toronto Area. The FHT is composed of 22 staff members: 6 primary care providers, 1 social worker, 2 registered nurses, 2 medical assistants, 3 diabetes educators, and 8 administrative staff.

### Sampling and Recruitment

Focus groups were conducted with community-dwelling patients with CCDD to learn what kinds of health and service issues were important to participants, what information should be collected, and how it could be collected in order to meet their needs. Purposive criterion sampling [26,27] was used to identify community-dwelling patients with CCDD to participate in this phase of our study. Purposive sampling is an appropriate approach for interpretive description studies like ours [24].To be included, focus group participants had to (1) have been identified as a patient with CCDD (defined as individuals with one or more health conditions that are difficult to manage), (2) be a patient at the FHT, (3) have the ability to give informed consent, and (4) understand and speak English. Approximately one third of the 5000 FHT patients fell into our definition of CCDD. Eligible participants were identified with the help of FHT staff. Recruitment posters with eligibility criteria, researcher contact information, and a brief description of the study were posted in the designated waiting area of the FHT as well. Participants provided consent to FHT staff to share their contact information with the research team and/or participants contacted research team staff directly to be included in the study. In a couple of cases, the patient was accompanied by their caregiver who expressed interest in participating and/or was required to attend to provide assistance to the patient. We did not originally intend to include caregivers, but those who expressed interest in participating were invited to attend.

### Procedure

Focus groups took place between November and December 2013. Between 6 and 9 participants were assigned to each focus group based on availability. After providing consent, all participants filled out a “participant information sheet”, which was used to collect data on age, gender, country of origin, and chronic illness profile. The catchment area of the FHT serves a diverse population of high and low income residents, and so we anticipated capturing a diverse group with regard to socioeconomic status. We did not feel the need to formally gather socioeconomic status data such as occupation or household income. Focus groups were semistructured around the questions listed in Textbox 1.

In addition to these questions, participants also had the opportunity to try out an example of a mobile monitoring system using a tablet, after which participants were asked: “What was it like answering questions using a tablet? What did you think about the content and wording used in the questionnaire that was downloaded onto the tablet?”

Focus groups lasted between 90 and 120 minutes and were audio recorded and transcribed by an external source. Transcripts were checked by the lead author (CSG) for accuracy. In addition to answering questions, participants were also presented with an example of an eHealth mobile app. Participants were invited to discuss whether a tool similar to the example provided might meet their needs and what types of challenges/barriers they may experience in using this type of technology.

Focus groups were conducted until new data resulted in only minor variations on identified themes in the codebook (ie, thematic saturation) [28,29]. Analyses of the first three focus groups generated a set of themes that were unchanged by the fourth focus group. As such, we were confident that nothing new could be learned from additional focus groups. An inductive analytic process that seeks thematic saturation is appropriate for interpretive descriptive research designs.

**Focus group questions.**

1. We are interested in understanding your experience in the health care system.

- Can you share with us the things that are important to you as a receiver of health care services?
- What can be done to improve things?

2. It is important for the health care system to gather information from you to better understand you and improve your care.

- What type of information should be collected from you?

3. How can health providers (or you as a person who uses health care) use technology to collect this information?

### Data Analysis

Inductive analysis was conducted concurrently with data collection through the identification, discussion, and notation of prominent themes between the two researchers conducting each focus group, generating a preliminary codebook that was applied to one focus group transcript by three researchers (CSG, DM, and CC). The prominent themes were discussed by the research team, and the codebook was revised.

Using the revised codebook, 2 researchers (CSG and DM) independently coded all transcripts using QSR NVivo 10 software. After each transcript was coded, the 2 researchers compared coding and reached consensus on all codes, modifying the codebook and codes applied to the transcript to reflect the consensus that was reached. For example, there was a discrepancy between how the concept “patient as expert” was coded by the 2 researchers, mainly revolving around whether patients viewed themselves as an expert in their care or perceived that the provider viewed them as an expert in their care. After reviewing a second and third transcript, the researchers came to a consensus that the concept should include both ideas (self-perceived and perception of the provider viewing the patient as an expert). The codebook was then modified and transcripts re-coded to reflect the new definition of the code.

This process was followed for each of the four transcripts. By the third and fourth transcripts, there were few discrepancies between the 2 researchers, demonstrating reliability of the thematic coding. The 2 researchers identified emerging subthemes through the coding process that are included in the findings. For example, the code “patient identified area of importance - communication” code applied to communication between patient and provider, between providers, and could include multiple forms of communication (ie, in person, telephone, electronic). These subthemes were identified by the 2 coders through the coding process to tease out the broad concept of communication. The coded data were next analyzed to identify relationships between codes. A table was created to demonstrate coding overlap, which was discussed and agreed on by the entire research team. This table informed the creation of an illustrated framework that demonstrates the connections between themes and subthemes. The framework is presented in the results section of this paper. The use of visual tools like our framework are recommended as part of the interpretive description approach [24] and help us to clarify how our concepts are related to each other.

In order to test the trustworthiness of the data, all focus group participants were given the opportunity to review the findings and provide feedback to the research team. Findings were presented in terms of concepts and themes representing the entire sample. This is an appropriate approach to participant validation for an interpretive descriptive study [23]; 6 of the 14 participants were amenable. The findings summary was mailed along with a feedback form and self-addressed and stamped envelope for the participants to fill out and return. Three responses were returned and confirmed that findings reflected their experiences and those discussed in their respective focus groups. Debriefing activities like this serve to support the credibility and trustworthiness of the data analysis [23,30]. It should be noted that one respondent identified additional subconcepts within the codes that were mentioned in their focus group, but that the participants felt were not evident in the summary. The subconcepts were reflected in the more detailed analysis used by the research team and as such were still captured in the analysis.

## Results

### Participants

The focus groups were conducted with patients with CCDD (n=10), caregivers (n=2), and those who were both caregivers and patients with CCDD (n=2). Patients included in the focus groups reported having multiple chronic illnesses including diabetes, chronic pain, osteoarthritis, osteoporosis, anemia, cardiac conditions, glaucoma, and mental illness. The average age of participants was 64.4 years; 9 participants were female, and 11 were born in Canada. While education level, socioeconomic status, and technological aptitude were not formally captured, these data were captured through researcher observation as well as through the information shared by participants during the focus groups. All participants were able to read and understand the consent form, which suggests at least a moderate literacy in English. Through the focus group conversations it was made clear that nearly half (n=6) held professional jobs that would require at least some post-secondary education. Most participants expressed that they were comfortable with computers and smartphones when they were presented with a device. Four participants made it clear that they were not as comfortable with these forms of technology, but only one participant did not attempt to engage with the sample device provided at the session.

Each focus group had between 2 and 5 members. Although our original aim was to have between 6 and 9 participants as suggested in the literature [31], but there were a number of last minute dropouts mainly due to illness. The timing of the dropouts did not allow for rescheduling within project timelines, and we did not wish to further burden patients by asking them to return. Hence, focus groups were conducted as per the original schedule. While the concern with low numbers in focus groups is a lack of adequate discussion [31], this was not a problem in any of the focus groups. Given that we reached thematic saturation (described above), the research team determined that additional groups with more participants were not required. Quotes from participants are identified by the focus group in which they participated.

### Important Issues for Patients With Complex Chronic Disease and Disability Receiving Health Care Services

#### Patient-Provider Interactions

Participants assigned high importance to their interactions with their primary care providers at the FHT, specialists, pharmacists, nurses, and health care administrators. Of importance to participants was the need for open, ongoing, two-way communication between themselves and their providers, particularly around test results:

I also need to know the results of tests when they happen. I need to know them…Like I need to look back and say this is what your test did, this is what it revealed, and this is what it means for the futureAnd if something is prescribed for me, why am I getting it or why is [my spouse] getting it, and what’s it supposed to do? And if it doesn’t do it, what do we do?

FG 1

Some participants noted that this timely feedback could help them to manage anxiety they were experiencing regarding their health:

So I go down and get the ultrasound [to check a lump]. And it was a good 5, 6 days before we get the information. It turned out to be nothing. But in those 5 days, I’m sitting there thinking, you know, have I got it [cancer]…It really works on your mind, you know.

FG 2

Participants did not just want to share information back and forth, but they wanted that exchange to be of high quality. Participants wanted an open “dialogue” with their providers in a space where they felt “heard” by a provider who was “taking time” to respectfully listen:

I don’t care if it’s on the phone, in-person, just make the time. Don’t rush us out the door like we’re a bloody number. We’re not on the slab, you know. We’re not a piece of meat. Listen to us, deal with us. Don’t push us out the frigging door because you’re not helping us like that.

FG 3

However, participants were weary of having to repeat themselves to different providers and of feeling as though they needed to “start from scratch” with every new provider they saw. Participants saw this as an issue that could be addressed through better information sharing between providers. Some participants suggested that improved patient information sharing between providers could be a proxy for ongoing relationships with a single provider that knew the patient’s history.

#### Provider-Provider Interactions

As might be expected, patients with CCDD tend to have multiple providers. Coordinating care between these providers was identified by participants in all focus groups as an ongoing problem in their care with regard to ensuring appropriate referrals, medication management, visits to the hospital, and overall coordination of care. One story provided by a participant describes the communication breakdown between a hospital and primary care provider:

That hospital did not notify [my primary care doctor]…I got out of the hospital and [my primary care doctor] said to me, “What happened?” I said, “Well, I don’t know what happened but I had to have bowel surgery.”…They did not give her any info on me. And she’s my family doctor.

FG 3

One participant noted a key issue with the lack of communication is that no one was looking at them holistically, stating:

…what happens is you have…somebody who looks at your hand, somebody who looks at your head. And nobody connects the whole thing together.

FG 4

An important issue raised by participants was identifying a single provider who had responsibility for the management of their information. Participants saw their primary care provider as being the “gatekeeper” of their information and as being responsible for having comprehensive information gathered from all providers:

If they’re specialists, they have limited knowledge and they don’t need to know about everything else about you. And I think it’s important that the family doctor communicate with the [specialist]. That the family doctor should be the gatekeeper of your charts and your data.

FG 4

And it’s true for all of us, if we don’t have a primary care physician who’s coordinating and navigating all of that, and helping us to understand what it is, then we’re off…through the system.

FG 1

Participants also wanted to know when their providers were communicating with each other, demonstrating a desire to be a part of the care process:

It’s done as though we’re not really a part of it. So until the second doctor gets back to us with an appointment, we have no knowledge whether the first contacted them or not. So it’s like if it’s supposed to take 4 months, we have to wait 4 months. And if it goes to 5 months, 6 months, then we might find out that they never did it. We’re left out of the equation.

FG 3

#### Access

A third key issue for participants was access to needed health care services, specialists, and treatments (mainly medications). Analysis revealed that access issues were often related to the patient-provider interaction issues identified above. Participants reported waiting to hear back from providers, to see providers (ie, in waiting-rooms), and for hospital beds. One caregiver thought that keeping patients waiting for a long time showed disrespect towards patients:

I want my time valued. I don’t want to sit in an office with my partner who is pretty hard of hearing, not deaf, and has complex health problems, watching him get more and more uncomfortable. And actually I’m very happy with the fact that here they’re seen promptly and it’s organized promptly. And that tells me that people respect me. That kind of respect is incredibly important.

FG 1

Participants reported that limited mobility made transportation to and from appointments challenging and that the costs associated with uninsured services impeded access:

But the transportation, my issue was that when you go to a specialist, you want to be able to go to someone who is on [public transit] because parking is an arm and a leg everywhere you go. And if they keep you waiting then you’re paying for 3 hours of parking.

FG 4

I don’t want them to say, ‘Well, here’s a list of practitioners that you should contact and get some…’ Like excuse me, I can’t afford $120 a visit. I’m retired now. So I would really like people to ask what kind of benefits do you have, and have a real list. You know, if I can afford it, if it’s covered, great. But if not, what resources are there for me beyond this relationship that I can avail myself of. Because to tell you the truth, and [spouse], my partner, that gives us a sense of hope and possibility. And we all need a sense of hope and possibility.

FG 1

A few participants shared concerns regarding inappropriate access, noting that they did not want to use scarce resources unless absolutely necessary. The fear of inappropriate use could actually deter patients from using services that may be needed, as illustrated by a story shared by one participant:

I had pain in my chest and in my jaw after I did some exercise. And I knew that these were no-no’s …So I had to call the health line because they were the only people I could talk to. And when I described what I was feeling to the woman on the phone, she said, ‘You go to [Toronto hospital] immediately. And you go to the emergency.’ And I went there and they hurried me into a room where they kept me hooked up to all kinds of things all day long. And at 5:00 in the afternoon, they gave me dinner and they sent me home. And I felt relieved but I was also embarrassed because I was not having a heart attack. And I used up some precious time and tests and materials and space and doctors. So maybe the next time I feel that, I won’t call. And maybe I will have a heart attack!”

FG 1

### Patient-Centered Approach

Participants identified the need for a patient-centered approach to their care. As suggested in patient-centered care literature, participants described wanting to be treated as whole persons, to feel as though they are seen as experts in their own care, and they identified the need for a strong, ongoing relationship between themselves and their health care providers built on trust:

I had someone before who looked at me, pegging me immediately as someone like her mother…But you know, like no, I’m not your mother…You really need to start with respect. We all deserve respect. Don’t have preconceived notions. Start with respect. Look at the whole person and really listen.

FG 1

But also at a certain age, you do have a background of experience that says, you know, this is how your body is and you tend to swell up when you eat salty food. [laughs] So this is what happened…So I know these things.

FG 4

It’s my life and my health here. Make me a part of it.

FG 3

### Needs Framework for Community-Dwelling Patients With Complex Chronic Disease and Disability

**Figure 1:**
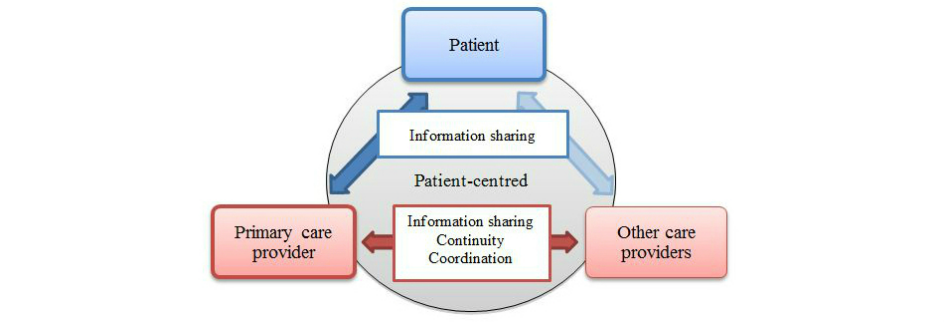
Needs framework for community-dwelling patients with CCDD in our study.

The health care needs identified by the participants in our focus groups can be conceptualized using a relatively simple framework (see Figure 1). This framework helps to visualize the relationship of the different needs and to identify areas where we could focus our eHealth technology development to be of most use to our CCDD patients. We depict the information sharing, communication, and access using arrows. Placing the patient at the apex of the relationship is intended to support the notion of patient-centeredness, which was important to participants in our study. The arrow between patient and primary care provider is emphasized compared to the arrow between the patient and other care providers, reflecting participants’ focus on their communication with their primary care providers and their feelings that their primary care providers are the central coordinators of their care. The arrows between providers are intended to reflect the need for bi-directional information sharing, continuity, and coordination identified as important to our participants.

### Information Sharing to Improve Care Using eHealth Tools

Focus group participants were asked about what kinds of information sharing would best support their needs. With regard to information exchanged between themselves and their providers, participants identified wanting to share medical and medication history, information regarding their symptoms and other health outcomes, and experiences with care. In terms of provider-to-provider information sharing, the emphasis was on sharing of medical and medication history as a means of improving coordination and continuity.

Participants saw significant potential for eHealth technologies to support the needs they had identified, summarized in Table 1.

**Table 1:** Supporting our CCDD patient needs using eHealth technologies.

eHealth app	Purpose	Quotes
Patient-provider information sharing	Monitoring symptoms by provider and self- monitoring by patient	*…anything that can help replace another visit to the doctor or an easy way to be monitoring a person who’s just come out of hospital at home, I think that it is so important.* [FG 1]
		*You could set this up to keep track of just how much you’re progressing or how much you’re regressing.* [FG 2]
	Patient accessing medical history	*But I’d want to know the results of the test.* [FG 3]
Provider-provider information sharing	Fast easy access to patient medical history	*I think the communication between each doctor would be a lot faster [using eHealth]. Like you’d have the patient file. They can each access it.* [FG 3]
	Coordination	*…if she was let go from the hospital, [the social worker] would have had all that information on the tips of their fingers—How is she going home and all, are we going to make something accessible, is the volunteer going to take her down? …Who is going to be at your home? Who is going to feed you? Do you want Meals on Wheels?* [FG 4]
	Continuity	*But besides that, it’s in print right in front of the doctor. She can read it and know it’s there, and she can recall it rather than, you know, talking on the phone with someone for 5 minutes and only taking in half of what the person said.* [FG 2]

### eHealth Tradeoffs

While participants were excited about the potential for eHealth to support their ongoing needs, they also identified a number of concerns with using eHealth tools. Participants expressed concerns regarding privacy and data security, accessibility (visual or motor impairment issues affecting the use of smartphone and tablets), the loss of necessary visits, increased social isolation, a new burden for overstretched providers, downloading responsibility onto patients for care management, entry errors, training requirements, and potentially confusing interfaces. Many of the anticipated challenges were related to participant-identified advantages, suggesting that the selection and design of eHealth applications may warrant cost-benefit analysis and awareness of trade-offs.

For example, participants liked the idea of ongoing monitoring and avoiding unnecessary physician and hospital visits, but some expressed concerns that the use of eHealth technologies may displace necessary in-person visits or contribute to isolation:

Like for people who are like bedridden and can’t get out, and you know, get their Meals on Wheels and stuff like that. If [eHealth monitoring is] the only contact that they’re going to have, that’s going to cut them off even more from society.

FG 4

Additionally while several participants called for wider sharing of patient information between providers, privacy and information security concerns were raised by others. Interestingly, a number of participants identified that the desire to have information shared easily, trumped their desire for privacy:

There are reams of x-rays and EKGs or ECGs. Stuff is sitting in doctors’ offices. Therefore if I have to go to a new doctor for whatever the reason, I want them to have it all. Short and sweet. I don’t care how. And I don’t want it to be my decision.

FG 1

I would like any health care professional, a doctor, whether it’s a specialist or a GP, be able to access that information.

FG 4

## Discussion

### Needs of Our Patients With Complex Chronic Disease and Disability

Our findings suggest that patients with CCDD at our FHT have a number of important care needs, among them being the need for improved communication and interactions between (1) themselves and their providers (both primary care and specialist providers), and (2) their different providers. Improved interactions between providers was also seen by participants in our study as a means to improve the coordination and continuity of their care. Our findings also highlight the need for these interactions recognizing the patient as a whole person and as an “expert” in their own care: concepts that are consistent with principles of person-centered approaches to care.

Findings from our study resonate with a previous study conducted with a similar patient population, but in an in-patient setting. Kuluski et al [32] conducted a qualitative study to help better understand the care needs and experiences of complex in-patients at a continuing complex care hospital in Toronto. The research team interviewed 116 patients who identified the need for improved communication with their providers and improved coordination of care (through supported transitions and more comprehensive patient assessment). A prominent theme in this study was the need for respectful interactions between providers and patients. Although the Kuluski et al study was conducted with in-patients, there are a number of similarities between the participants in this study and our own; the average age of participants was 63, mostly female, with multiple morbidities.

A number of ways that eHealth technologies could support the health care needs were identified by participants in our study. A key focus for our participants was the role eHealth technologies can play in supporting interactions between patients and providers and between different providers.

### Developing eHealth Tools to Support Patient-Provider Interactions

Provider-patient interactions identified as important by participants involved patients sharing information back to providers regarding symptoms (monitoring) and patients’ being able to access their health information. These communication pathways may be facilitated through the use of electronic health records, telemedicine or telehealth care, and technologies to support patient monitoring, sometimes referred to as telemonitoring [33-35]. Prominent eHealth tools that may be useful include:

- Electronic Medical Records (EMRs): Software used at a single organization to collect, manage, and store patient health information (replacing old paper files) [20].
- Electronic Health Records (EHRs): Electronic systems that allow for the sharing of health data across different providers and health organizations [36] (see also [37]).
- Electronic Patient Health Records (PHRs or EPRs): Electronic applications that allow patients to access, manage and share their health information [20,38].
- Telemonitoring and Web applications: Electronic systems that allow patients to remotely transfer data to one or more health care providers [17].
- Web-based resources: These may include health information websites and online peer-to-peer support groups [35,39].

Electronic PHRs and telemonitoring systems can offer opportunities for improved continuity of care, efficiency, decision-making support, and greater partnerships between patients and their caregivers and providers [38]. One qualitative study conducted by Woods et al found that patient access to their EMR information improved patient-provider communication by (1) enhancing in-person communications, (2) helping patients to remember what was said at in-person visits, (3) helping patients to prepare for future appointments, and (4) helping patients to coordinate with their other providers [40]. Accessing EMR information was also found to improve patient self-management and supported shared decision-making between patients and providers.

There have been a number of studies examining the use of eHealth technologies to monitor patients on an ongoing basis. Two recent systematic reviews found that eHealth-supported monitoring can improve outcomes for patients with chronic illnesses (including diabetes, asthma, hypertension, and cardiac obstructive pulmonary disease) [16,34]. One of the reviews also found evidence that monitoring symptoms helped patients with the self-management of their care, leading to improved health benefits, patient satisfaction, and reduction in physician visits and appointment times when compared to standard care [16]. Whether eHealth-supported monitoring will improve outcomes and self-management for CCDD patients is yet to be seen given that there are few tools designed for this population. We might expect, however, that outcomes identified above may be highly beneficial for participants in our study who identified difficulties with self-management and with accessing providers due to transportation or cost issues.

### eHealth Tools to Support Provider-Provider Interactions

Participants in our study identified a number of problems associated with a lack of communication between their multiple providers. Sharing patient information, for instance through a commonly accessible EMR, was identified as an important step towards improving interprovider communication and as means to improve the continuity and coordination problems participants experienced. There have been many calls in the literature to use EHRs, EMRs, and PHRs to support integration, care coordination, and continuity [36,41-45]; however, not all electronic systems are created equal.

EMRs may be useful for intra-organizational coordination and continuity but limited when it comes to supporting interorganizational communication. A qualitative study of physician use of EMRs in the United States found that while EMRs were able to facilitate within-office care coordination, the lack of standardization and inadequate operational processes limited their capacity to encourage coordination between different health care organizations [44]. What would be more appropriate, particularly for participants in our study with CCDD resulting in their having multiple providers at different organizations, would be an EHR [36] that houses patient information at a system level rather than at a single organization. Given that CCDD patients experience social, as well as medical complexity [4], there is the added challenge of making EHR data available to social service providers outside of health care, such as social workers, who may be important care team members for patients with CCDD. The need to expand our definition of providers in the context of CCDD patients will undoubtedly raise new challenges with regard to data security and privacy. Determining which providers need access to what types of information and how that access is granted will need to be addressed.

### Weighing eHealth Tradeoffs

An important finding in our study is the concern of participants regarding the adoption and use of eHealth technologies. Issues of shifting responsibilities, changing patient-provider interactions and relationships, and privacy concerns identified by participants have been noted in the eHealth literature [36,39,46,47]. However, similar to findings in our study, one study overviewing patient input into the development of a new EHR system in the United States found that the patient-perceived potential benefits of an EHR system outweighed patients’ concerns regarding privacy and security [48].

In addition to the potential issues with eHealth identified in our focus groups, there have been some studies to suggest that increasing patients’ access to their medical information and engaging them in monitoring could actually increase anxiety [35,40]. As CCDD patients will often experience mental health challenges [4], an impact on anxiety as a result of using the tool may be a particular concern when developing monitoring technologies. While increased patient anxiety was not raised in our focus groups, primary care providers identified this as a concern through informal discussions with the research team. In designing our tools, we will ensure that we include patient debriefs and monitoring for increased anxiety so unintended adverse events can be avoided.

### Limitations

A potential limitation is that participant opinions may be shaped by their perception of what is socially acceptable, which is a limitation for most qualitative studies, particularly focus groups in which participants may feel pressure to share only opinions they feel are shared by the group. Another limitation was the small size of the focus groups; in one instance, a group contained only 2 individuals. While we were still able to maintain a meaningful and rich conversation (as noted in the methods section), more individuals in the room may have spurred additional conversation that may have elicited additional concepts that were not captured. However, reaching thematic saturation suggests no new topics were likely to arise even with additional participants. It is also possible that fewer participants in the focus group allowed for more in-depth discussion and could as such be considered a strength of the study. The use of appropriate study methodology and rigorous analysis approach is another notable strength.

One important limitation may be that many participants had noted an existing comfort with mobile and computer technologies. It is possible that a less technologically savvy group may not have been so positive about the potential for eHealth technologies to help support their needs. However, one participant refused to use the technology, and a few others were not as comfortable with the technology, and their concerns were reflected in our study.

### Implications for Development of Our Tools

These study findings provide us with important groundwork to start the development of eHealth tools to support community-dwelling patients with CCDD. We are encouraged that the participants in our study perceive that eHealth technologies could be beneficial to supporting their needs in primary care settings. Our focus group participants identified that they require improved patient-primary care provider communication, improved interprovider communication, and that while eHealth technologies can offer a number of benefits, there are potential tradeoffs that researchers and developers should take into consideration. We will begin with a focus on developing telemonitoring to support ongoing patient-provider interaction and patient self-management. As our health care system in Ontario is far from having an integrated EHR, our monitoring tools will include a portal system to allow patients to share data with multiple providers.

A key challenge we, and many others working in eHealth, face are the challenges in supporting interprovider communication. In particular are the barriers associated with creating a commonly accessible EHR such as lack of standardization of clinical information, patient concerns over security and privacy, provider concerns over legal liability, and costs [36]. Given these barriers, a first step forward may be simply providing patients with CCDD mobile access to their medical records that they could then share with their multiple providers at the point-of-care or by giving providers access to a Web-based portal. Although, we could design a tool that allows for multiple provider access, implementing this strategy in a fractured system, as is the case in Ontario and much of Canada, is likely to be a challenge. Through piloting we will determine the feasibility of this approach and identify other options for improving interprovider communication to support patients with CCDD.

In order to avoid the potential pitfalls of eHealth technologies identified by our participants, we will adopt a user-centered design approach to develop our tools, allowing us to design and implement our tools in partnership with patients. User-centered design fits within the broader design evaluation approach used for our project and supports our aim to keep patient and provider users heavily involved in the full development process. In keeping with the user-centered design methods, we anticipate multiple iterations of our tools that will be reviewed by both patients and providers at each step. While the literature identifies the potential and realized benefits of eHealth tools, many of these tools and studies focus on patients with single diseases. Our tool will be addressing a notable gap in eHealth technology through the development of patient-centered tools specifically for patients with CCDD.

### Conclusions

From our patients’ perspectives, there is a significant potential for eHealth tools to support patients with CCDD in community and primary care settings through enhancing two-way communication between patients and providers, and care coordination and continuity through improved interprovider communication. However, we need to be wary of the potential downfalls of adopting eHealth technologies and pay special attention to patient-identified needs and concerns. We are thus encouraged that the patient-centered eHealth tools we intend to build will be able to address the many challenges faced by patients with CCDD at our particular setting. As we move into the piloting and evaluation phases, we will seek to roll out the tool more broadly to other team-based primary care settings. The strength of our approach is in using patient-identified needs to drive tool development, allowing us to build patient-centered tools and support patient-centered care more broadly.

## Abbreviations

CCDD: complex chronic disease and disability
EHR: electronic health record
EMR: electronic medical record
FHT: family health team
PHR: personal health record
